# Preoperative neutrophil/lymphocyte ratio predicts overall survival but does not predict recurrence or cancer-specific survival after curative resection of node-positive colorectal cancer

**DOI:** 10.1186/1471-2407-13-442

**Published:** 2013-10-01

**Authors:** Lucy Jankova, Owen F Dent, Charles Chan, Pierre Chapuis, Stephen J Clarke

**Affiliations:** 1 Bill Walsh Translational Cancer Research Laboratory, Kolling Institute of Medical Research, Royal North Shore Hospital, St Leonards, NSW 2065, Australia; 2 Sydney Medical School, University of Sydney, Sydney NSW, Australia; 3 Northern Translational Cancer Research Unit, Sydney NSW, Australia; 4 Department of Colorectal Surgery, Concord Repatriation General Hospital, Concord NSW, Australia; 5 Department of Anatomical Pathology, Concord Repatriation General Hospital, Concord NSW, Australia; 6 Department of Medical Oncology, Royal North Shore Hospital, St Leonards NSW, Australia

**Keywords:** Colorectal cancer, Neutrophil/lymphocyte ratio, Survival, Prognostic biomarker, Competing risks Cox regression

## Abstract

**Background:**

The preoperative ratio of neutrophils to lymphocytes (NLR) has been proposed as a marker of poor outcome in patients having a resection for colorectal cancer (CRC). This study investigated the association between NLR and overall survival, cancer-specific survival and recurrent cancer in patients who had a potentially curative resection for node-positive CRC.

**Methods:**

Data on 322 patients were drawn from a prospectively recorded registry operated on between 1999 and 2007. Analyses of survival involved the Kaplan-Meier method, Cox regression and competing risks Cox regression.

**Results:**

Increasing NLR as a continuous variable was independently though weakly associated with diminishing overall survival after adjustment for other prognostic variables (HR 1.06, 95% CI 1.01-1.11, *p* = 0.013). Receiver operating characteristic analysis to dichotomize NLR as a predictor of overall survival yielded relatively poor sensitivity (55%), specificity (66%) and positive predictive value (56%, CI 47%-64%). Competing risks regression also showed that NLR was not independently associated with recurrence at any site (HR 1.04, CI 0.97-1.11, *p* = 0.241) or CRC-specific mortality (HR 1.02, CI 0.92-1.12, *p* = 0.782) but was associated with non-CRC mortality (HR 1.09, CI 1.03-1.15, *p* = 0.004).

**Conclusion:**

In patients with stage C tumor the weak link between NLR and overall mortality was not specific to CRC but apparently arose because patients with an elevated inflammatory status preoperatively were likely to progress to earlier death but not necessarily because of their cancer.

## Background

Inflammation has been associated with the development of numerous malignancies including colorectal cancer [1]. In addition, evidence of an ongoing systemic inflammatory reaction, in particular the modified Glasgow Prognostic Score (mGPS), has been shown to predict earlier tumor relapse and mortality in operable colorectal cancer patients [2,3]. The mGPS is a 3-point scale and is derived from measurements of serum albumin and C-reactive protein (CRP) concentrations. However, CRP is not routinely measured and thus is generally not available in datasets of well-characterised historical cohorts.

The ratio of circulating neutrophils to lymphocytes (NLR) is another indicator of systemic inflammatory response and has been proposed as a routinely available preoperative indicator of prognosis in patients undergoing resection of primary colorectal cancer (CRC) [4-7] (Table 1). The origin of this suggestion was a study of serial postoperative observations of neutrophils and lymphocytes which showed that the ratio of these two factors was an effective indicator of the intensity of physiological stress in ICU patients after CRC resection or surgery for abdominal sepsis or medical treatment of severe sepsis or septic shock [8]. Subsequently, studies of patients with primary CRC have reported a statistically significant association between preoperative NLR and overall survival [4,7,9], although this association was not found in one study [10]. Associations have also been reported between NLR and recurrence-free or disease-free survival [6,9,11,12] but not cancer-specific survival [7,10]. These studies all examined both simple bivariate associations between NLR and survival and multivariable models including other known predictors of outcome, though the association with overall survival did not persist in a multivariable model in two cases [4,7] and another study yielded the surprising finding of a multivariable association between NLR and cancer-specific survival despite no bivariate association [5]. The variability in findings among these studies is perplexing and further research on NLR and prognosis is clearly necessary. It is of particular concern that all of the above studies used an outmoded method of analysing the outcome measures of cancer-specific survival. None employed competing risks methods which are free of the biases introduced by traditional methods [13] and are now regarded as the most appropriate techniques for analysing such data [14,15].

**Table 1:** Reports on the association between NLR and patient survival after resection of primary colorectal cancer

**Report**	**Study design**	**Site**	**Stage and number of patients**	**Exclusions**	**Haematology measured**	**Pre-op RT or RT/CT**	**Adjuvant CT**	**Minimum follow-up time of surviving patients**	**NLR variable**	**NLR Significant in bivariate analysis**	**NLR Significant in multivariate analysis**
Walsh 2005 [7]	Retrospective	Colorectal	A: 30	None specified	For stages A-D:	3 patients	Not	24 months	Binary	OS: yes	OS: no
			B: 80				specified		< 5 vs. ≥ 5	CSS: yes	CSS: no
			C: 65		Pre-op						
			D: 26		For unresected:						
			unresected								
			29		during diagnosis						
			Total 230								
Leitch 2007 [10]	Retrospective	Colorectal	I: 22	Infection or	Not specified	11	Not	36 months	Binary	Stages I-III	Stages I-III
			II: 62	inflammatory		patients	specified		< 5 vs. ≥ 5	OS: no	OS: no
			III: 65	conditions						CSS: no	CSS: no
			IV: (liver mets) 84							Stage IV	Stage IV
										OS: no	OS: no
			Total 233							CSS: no	CSS: no
Ding 2010 [12]	Retrospective	Colon	IIA: 141	Adjuvant	< 1 week	Not	Not	Not	Binary	RFS: yes	RFS: yes
				CT	before	specified	specified	specified	≤ 4 vs. > 4		
				Multiple	resection					The precise	
				primaries,						definition	
				polypolsis,						of RFS in	
				HNPCC,						respect of	
				infection or						censoring	
				haematological						is not	
				disorders						specified	
Liu 2010 [5]	Retrospective	Rectum	I: 17	Synchronous or	Not specified	Not	All stage	Not	Binary	CSS: no	CSS: yes
			II: 59	metachronous		specified	III or IV	specified	<2 vs. >2		
			III+IV: 47	cancer.					Not		
			Total 123	Lost to follow					specified		
				up					when		
									exactly 2		
Hung 2011 [9]	Retrospective	Colon	II: 1040	Adjuvant	Before	Not	Excluded	46 months	Binary	OS: yes	OS: yes
	based on			CT	resection	specified			<5 vs. ≥5	DFS: yes	DFS: yes
	tumor									DFS not	
	registry									precisely	
	data									defined	
Kwon 2012 [4]	Retrospective	Colorectal	I: 13	Emergency	1 day before	Not	150	Not	Binary	OS: yes	OS: no
			II: 91	Surgery.	resection	specified	patients	specified	< 5 vs. ≥ 5		After
			III: 88	Death < 30							adjusting for
			IV: 8	days after							platelet/
			Total: 200	surgery.							lymphocyte
				Infection or							ratio
				inflammatory							
				conditions							
Chiang 2012 [11]	Retrospective	Colorectal	Curative	Anal cancer.	Pre-op	124	73	11.6	Binary	DFS: yes	DFS: yes
			only	Primary site	undefined	patients	patients	months	≤3 vs. >3		
			undefined.	indefinite.						DFS not	
			Composite	Synchronous		not				precisely	
			TNM	colon & rectum.		excluded				defined	
			stage not								
			given;	Complicated							
			Total	presentations.							
			patients in								
			NLR survival								
			analyses = 3177								
Mallappa 2013 [6]	Retrospective	Colorectal	297 patients.	Inflammatory or	Pre-op	excluded	Not	Not	Binary	DFS: yes	DFS: yes
				haematological	undefined		specified	specified	< 5 vs. >5		
			Jass stage:	disorders.						DFS not	
			1: 90	Pre-op RT					Not	precisely	
			2: 71	Emergency					specified	defined	
			3: 58	resection.					when		
			4: 78	Non-curative.					exactly 5		
				Died ≤ 30 days							
				postoperatively.							

*NLR* neutrophil/lymphocyte ratio, *OS* overall survival.

*CSS* cancer-specific survival, *RFS* recurrence-free survival.

*DFS* disease-free survival, *PFS* progression-free survival.

*CB* clinical benefit, *TTLR* time to local recurrence.

*Mets* metastasis, *HNPCC* Hereditary non-polyposis colorectal cancer.

*Pre-op* preoperative, *RT* radiotherapy, *CT* chemotherapy.

As mentioned, the preoperative NLR has been proposed as a useful prognostic marker because it is based on inexpensive data acquired routinely and early during the investigation of patients for CRC and when taken together with pathological information from the operative specimen, it may also yield useful independent information on prognosis. Studies investigating this have been based on patients with various pathological stage mixes including TNM stage II only, [9] stage IIa only [12] and stages I to IV [4-6,10], in one case including patients with unresectable tumors [7]. In analysing the prognostic potential of a marker it is important to choose a patient pool to which the marker can most appropriately and productively be applied clinically. Patients with stage I CRC have an almost uniformly good prognosis whereas those with stage IV tumor invariably have poor outcomes and thus a pre-operative NLR is unlikely to provide prognostic information that could alter treatment in either of these patient groups, although it may predict response to therapy in stage IV patients [16,17]. It is more likely that NLR could provide additional prognostic value after potentially curative resection of II or stage III tumor. In particular, patients with stage III tumor form a heterogeneous group and there is a need for markers that can lead to more precise prognosis and hopefully differentiate between patients who may benefit from additional adjuvant systemic chemotherapy and those who will not. Although the role of NLR in stage II tumor has already been described [9,12], to our knowledge its ability to predict outcomes after potentially curative resection of lymph node positive CRC has not been investigated.

All prior reports on NLR in primary CRC have converted the continuous measure of NLR to a binary variable in analyses, in most cases using the cutting point of NLR < 5 versus ≥ 5 [4,6,7,9,10] as proposed by Zahorec [8]. However, various other cutting points have also been employed [5,11,12]. The point chosen to dichotomize NLR values could potentially have an important influence on the findings of a study. The threshold should not be chosen arbitrarily but should be determined by an objective optimizing technique which is relevant to the particular outcome under investigation, as different thresholds may be appropriate for different outcomes.

The aim of this study was to examine the association between preoperative NLR and tumor recurrence, overall survival and colorectal cancer-specific survival after resection of stage C CRC. A secondary aim was to investigate the optimal threshold for dichotomizing NLR as a predictor of survival.

## Methods

### Patient population

Information on patients having a resection for CRC performed by members of the Concord Hospital Department of Colorectal Surgery has been entered into a prospective computer database since 1971 [18,19]. The data set contains details of patient characteristics, comorbidity, presentation, investigations, surgical management, complications, adjuvant therapy, pathology and follow-up and has the approval of the South Western Sydney Health Area Ethics Committee. Patients described in the present study had a resection for stage C CRC between November 1999 and December 2007 inclusive. All resections were performed by specialist colorectal surgeons following a standardized procedure [20,21] and data acquisition and recording was supervised by a single surgeon (P.H.C.).

Patients were excluded if they had had a colorectal cancer previously or if they had adenomatous polyposis coli, ulcerative colitis, Crohn’s disease or if they had received preoperative chemoradiotherapy. Seven patients received postoperative radiotherapy but were not excluded. Pathological examination of the resected specimen followed a standard protocol [18,22]. Only adenocarcinomas (including mucinous and signet ring carcinomas) were included in the data set. Where multiple tumors were present, only the lesion with the most advanced stage was included. Tumor size was measured as the greatest surface dimension and dichotomized as < 5 cm versus ≥ 5 cm. Blocks were taken to demonstrate maximum direct tumor penetration of the bowel wall. Additional blocks were taken specifically to demonstrate the relationship between tumor and any adherent structure or tissue [23] as well as lines of resection and the free serosal surface [24]. Venous invasion referred to involvement of thick or thin walled veins, either within or beyond the bowel wall. When doubt existed as to whether a structure involved was a vein, a negative finding was recorded. An apical lymph node was defined as the most proximal of any nodes found within 1 cm of the vessel ligation at the apex of a vascular pedicle [25]. The proportion of involved lymph nodes was calculated as the number of positive nodes divided by the number of nodes harvested expressed as a percentage and was dichotomized at < 40% versus ≥ 40%. All pathology features analysed were looked for in every specimen and their presence or absence recorded explicitly and there were no missing data on any pathology variable. In analyses, all patient and tumor characteristics which were not natural binary variables were dichotomized in order to simplify comparisons of effect sizes between covariates in multivariable survival models. Tumors were staged according to the Australian Clinicopathological Staging System for colorectal cancer which accommodates sub-stages compatible with other clincopathological staging systems such as TNM [26]. Patients selected for the present study had tumors involving local lymph nodes but without distant metastasis (stage C) but not including any with frank tumor in a proximal, distal, circumferential or deep line of resection.

The NLR was defined as the absolute count of neutrophils divided by the absolute count of lymphocytes determined from the full blood count routinely taken within the week before resection. This information was not recorded in the prospective registry of patients but was available in hospital files from 1999 onwards, though was unavailable for 10 patients.

### Follow-up and assessment of survival and recurrence

Patients were seen at least six-monthly for the first two years after resection and yearly thereafter until death or December 31, 2010. Surveillance included clinical examination, sigmoidoscopy, a chest x-ray and serial CEA measurements. For rectal cancer a CT scan was performed annually as well as a colonoscopy, the latter especially in those patients who had initially presented with obstruction due to a stenotic tumor and in whom examination of the proximal colon had not been possible. For colon cancer, colonoscopy was generally repeated at 3 to 5 years following resection. Recurrence was defined as clinically or radiologically suspected or biopsy proven tumor in the pelvis, perineal scar or peritoneal cavity, or newly diagnosed distant metastasis. Cause of death was ascertained from the patient’s surgeon or family physician or hospital records or from a close relative or, in a small number of cases, from the national registry of causes of death.

Overall survival time was measured from the date of resection to the date of death due to any cause with times censored at last contact for patients who were lost to follow-up or who remained alive at the close of study in June 2012. Colorectal cancer-specific survival was measured from resection until the date of death due to colorectal cancer with times censored at last contact for patients who were lost to follow-up or who remained alive at the close of study. The survival times of patients who died of causes other than colorectal cancer were measured until the date of death and these patients were coded as having experienced a competing risk in regression analyses. Time to recurrence was measured until the date of diagnosis of recurrence except for seven patients who died of CRC but whose precise recurrence date was not known, in which cases the date of death was substituted. Times were censored at last contact for patients who were lost to follow-up or who remained alive and recurrence-free at the close of study. Patients who died without recurrence were classified as having experienced a competing risk in regression analyses.

### Statistical analysis

All analyses were conducted on the basis of intention to treat. Because of the markedly skewed distribution of NLR, associations between it and other covariates were assessed by the Mann-Whitney U test. Proportional hazards regression or competing risk regression was used to assess the effect of NLR as a continuous variable on survival time and also for comparisons of survival time between strata of binary variables. In multivariable modelling, all covariates having an association with survival with a Wald test *p* value < 0.1 were entered into an initial regression model which was then reduced by sequential removal of covariates with a *p* value of > 0.05, beginning with the highest *p* value until a provisional final model containing only covariates with a *p* value ≤ 0.05 was attained. Excluded variables were then reintroduced singly into this model but none achieved significance. The assumption of proportional hazards for the continuous version of NLR was assessed by inspection of Schoenfeld residuals, and for dichotomous covariates by examination of log cumulative hazard plots for parallelism and in no case was it materially violated in any variable included in a regression model. Possible interactions between NLR and other covariates were examined by introducing product terms singly into the final model but no significant interactions were identified.

Two different methods were used in an attempt to identify an optimal cutting point for NLR as a dichotomous predictor of overall survival time. The first was the conventional ROC curve method with death due to any cause as the outcome. The disadvantage of this method is that all patients remain in the calculations whether or not their survival times are censored. The second method, based on Kaplan-Meier curves and proportional hazards regression, does take account of censoring. NLR was first split at 0 to 1.49 versus ≥ 1.5 and Kaplan-Meier curves and the hazard ratio, 95% confidence interval and Wald *p* value were calculated. The cutting point was then raised in steps of 0.5 (0-1.99 vs. ≥ 2, 0-2.49 vs. ≥ 2.5, etc.) and the results recalculated at each step in order to identify the threshold giving the greatest separation of curves with the lowest *p* value. The same process was applied in both a bivariate and a multivariable model.

The level for two-tailed statistical significance was *p* ≤ 0.05 with confidence intervals (CI) at the 95% level. Analyses were performed with SPSS version 20 (IBM) and Stata release 12 (Stata Corporation, College Station, TX, 2011).

## Results

During the study period 1388 patients had a resection for colorectal cancer. Of these, 1011 were excluded because their tumor was not stage C; 12 were excluded because of previous CRC; 3 because of inflammatory bowel disease and 1 because of adenomatous polyposis coli. Of the 361 patients remaining, preoperative haematology results were not available retrospectively for 10 and 29 were excluded because they had received neoadjuvant chemoradiotherapy, leaving 322 for analysis. Characteristics of these patients are shown in Table 2.

**Table 2:** Clinical and pathology characteristics of 322 patients with stage C colorectal cancer and association between these characteristics and NLR

	**Total n = 322 n (%)**	**Median NLR**	**Mann- Whitney *p *value**
Male	179 (55.6)	2.7	0.942
Female	143 (44.4)	2.6	
Age ≥ 75 years	108 (33.5)	3.2	<0.001
Age < 75 years	214 (66.5)	2.5	
Rectal tumor	127 (39.4)	2.5	0.007
Colonic tumor	195 (60.6)	2.8	
Tumour size ≥5cm	137 (42.5)	2.8	0.014
Tumour size <5cm	185 (57.5)	2.6	
Mucinous or signet ring	34 (10.6)	2.9	0.433
Other adenocarcinoma	288 (89.4)	2.6	
Direct spread beyond muscularis propria	271 (84.2)	2.7	0.718
Not beyond muscularis propria	51 (15.8)	2.5	
Apical node involved	30 ( 9.3)	3.1	0.040
Not involved	292 (90.7)	2.6	
≥ 4 nodes involved	102 (31.7)	2.8	0.467
< 4 nodes involved	220 (68.3)	2.7	
≥ 40% of nodes involved	60 (18.6)	3.0	0.011
< 40% of nodes involved	262 (81.4)	2.6	
Poorly differentiated	66 (20.5)	3.0	0.014
Moderately or well differentiated	256 (79.5)	2.6	
Venous invasion	64 (19.9)	2.8	0.748
No venous invasion	258 (80.1)	2.7	
Free serosal surface involved	76 (23.6)	3.1	0.010
Not involved	246 (76.4)	2.6	
Adjacent structure infiltrated	23 ( 7.1)	3.7	0.021
Not infiltrated	299 (92.9)	2.6	
Postoperative chemotherapy	197 (61.2)	2.5	0.006
No postoperative chemotherapy	125 (38.8)	3.0	

Number, (%).

The distribution of neutrophils ranged from 1.7 to 12.8 with a mean of 4.7 (SD 1.8), a median of 4.3 and mild positive skewness (1.4). The distribution of lymphocytes ranged from 0.3 to 3.8 with a mean of 1.7 (SD 0.6), a median of 1.6 and was approximately bell-shaped with slight positive skewness (0.7). The NLR ranged from 0.7 to 28.5 with a mean of 3.3 (SD 2.7), a median of 2.7 and very marked positive skewness (5.0).

Among the 14 clinical and tumor characteristics examined, NLR was significantly higher in patients aged years 75 or older; for colonic tumors; for tumors ≥ 5 cm; when an apical node was involved; for tumors with ≥ 40% of nodes involved; for poorly differentiated tumors; when a free serosal surface was involved; when an adjacent structure was infiltrated by tumor, and in patients who had not received postoperative chemotherapy (Table 2).

At the close of the study in June 2012, 6 patients (1.9%) had died before discharge from hospital after their resection, 3 (0.9%) had been lost to follow-up after 5.9, 31.6 and 51.1 months respectively and 135 had died after a median of 34.7 months (range 0.7 to 138.3 months). Median survival time in the 191 patients who remained alive and were not lost was 82.2 months (range 34.3 to 146.8 months).

### Overall survival

A Cox regression model with NLR as the single, continuous covariate showed that overall survival diminished significantly as NLR increased (hazard ratio [HR] 1.11, 95% CI 1.06-1.15, p < 0.001) (Table 3). Other characteristics having a significant bivariate association with diminished overall survival were age ≥ 75 years, direct spread beyond the muscularis propria, apical node involvement, ≥ 40% of nodes involved, adjacent structure infiltrated by tumor, absence of adjuvant chemotherapy, poor differentiation, ≥ 4 involved lymph nodes, and involvement of a free serosal surface, however a multivariable model showed that the last three did not have significant independent effects (Table 3). It was concluded that increasing NLR was independently associated with diminishing overall survival after adjustment for other prognostic variables.

**Table 3:** Association between NLR, clinical and pathology features and overall survival

	**Number of patients**	**Number of deaths**	**Bivariate association HR, (95% CI), Wald*p***	**Multivariable association HR, (95% CI), Wald *p***
Neutrophil/lymphocyte ratio	322	141	1.11 (1.06–1.15) *p* < 0.001	1.06 (1.01–1.12) *p*= 0.013
Male	179	79	1.04 (0.74–1.45) *p* = 0.831	--
Female	143	62		
Age ≥ 75 years	108	75	3.32 (2.37–4.63) *p* < 0.001	2.15 (1.42–3.27) *p* < 0.001
Age < 75 years	214	66		
Rectal tumor	127	54	0.88 (0.62–1.23) *p* = 0.446	--
Colonic tumor	195	87		
Tumor diameter ≥ 5cm	137	62	1.12 (0.81–1.57) *p* = 0.490	--
< 5cm	185	79		
Mucinous or signet ring	34	19	1.35 (0.83–2.20) *p* = 0.224	--
No	288	122		
Direct spread beyond muscularis propria	271	126	2.02 (1.18–3.45) *p* = 0.010	2.04 (1.18–3.54) *p* = 0.011
No	51	15		
Apical node involved	30	18	1.80 (1.08–3.00) *p* = 0.009	2.03 (1.22–3.39) *p* = 0.007
No	292	123		
≥ 4 nodes involved	102	58	2.01 (1.43–2.81) *p* < 0.001	--
No	202	83		
≥ 40% of nodes involved	60	41	2.71 (1.88–3.91) *p* <0.001	2.52 (1.71–3.70) p <0.001
No	262	100		
Poorly differentiated	66	42	2.12 (1.47–3.04) *p* <0.001	--
No	256	99		
Venous invasion	64	33	1.44 (0.98–2.13) *p* = 0.066	--
No	258	108		
Free serosal surface involved	76	47	2.36 (1.65–3.35) *p* < 0.001	--
No	246	94		
Adjacent structure infiltrated	23	17	3.16 (1.90–5.25) *p* < 0.001	2.88 (1.71–4.86) *p* <0.001
No	299	124		
Postoperative chemotherapy	197	61	0.61 (0.26–0.51) *p* < 0.001	0.56 (0.37–0.85) *p* = 0.006
No	125	80		

**Figure 1:**
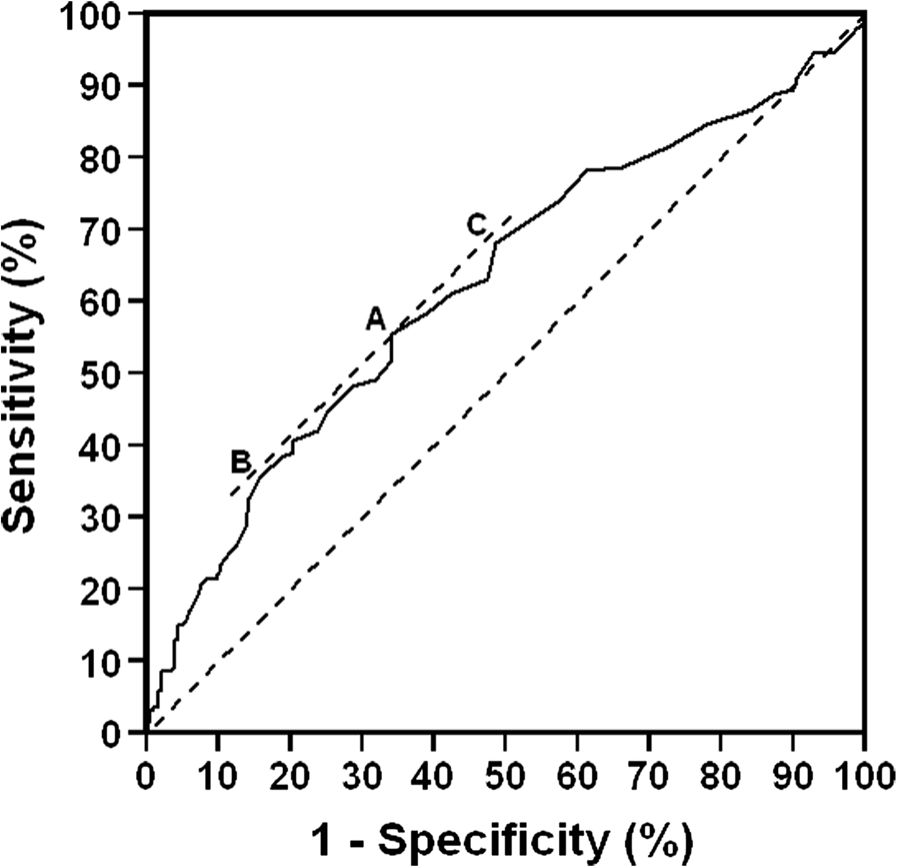
**ROC curve for the NLR as a predictor of death due to any cause.** The optimum threshold of 2.8 is indicated by A, at which point the positive predictive value was 56% (95% CI 47%-64%). B indicates a threshold of 3.8, at which the positive predictive value was 63% (CI 52%-74%). C indicates a threshold at 2.5%, at which point the positive predictive value was 52% (CI 45%-60%).

The results of the search for the optimal threshold for dichotomizing NLR in relation to overall survival time were unconvincing. The ROC curve method yielded an optimum cutting point at an NLR of 2.8 (Figure 1), though the ROC curve lay almost parallel to and not greatly distant from the null curve over the range from NLR = 2.4 to 3.8. At the optimum threshold of 2.8 the sensitivity was 55% (CI 47-64%), the specificity was 66% (CI 58-73%), the negative predictive value was 65% (CI 58-72%) and the positive predictive value was 56% (CI 47-64%).

Using the alternate method for fixing the threshold, the bivariate hazard ratio became significant at an NLR threshold of ≥ 2.5 (HR 1.96, CI 1.37-2.79, p < 0.001) and increased to 2.84 (CI 1.74-4.42, p < 0.001) at an NLR threshold of ≥ 7 but only 12 of the total of 141 deaths occurred to patients above this point (Table 4), indicating that high NLR defined by this threshold was an unsatisfactory predictor of death. From this analysis of survival time to death from any cause using two different methods it was concluded that no clearly distinctive optimum threshold could be identified.

**Table 4:** Association between overall survival and NLR at progressive thresholds for dichotomizing NLR

**NLR dichotomy**	**Patients number (%) n = 322**	**Deaths n = 141**	**Bivariate hazard ratio, (CI), Wald *p***
<1.5	30 ( 9)	13	
≥1.5	292 (91)	128	1.14 (0.64–2.01) 0.6584
<2.0	75 (23)	26	
≥2.0	247 (77)	115	1.40 (0.91–2.14) 0.1229
<2.5	138 (43)	45	
≥2.5	184 (57)	96	1.96 (1.37–2.79) 0.0002
<3.0	187 (58)	68	
≥3.0	135 (42)	73	1.88 (1.35–2.61) 0.0002
<3.5	257 (80)	101	
≥3.5	65 (20)	40	2.15 (1.53–3.01) <0.0001
<4.0	268 (76)	107	
≥4.0	83 (24)	50	2.07 (1.43–2.98) 0.0001
<4.5	273 (85)	110	
≥4.5	49 (15)	31	2.07 (1.39–3.09) 0.0004
<5.0	287 (89)	117	
≥5.0	35 (11)	24	2.48 (1.60–3.86) 0.0001
<5.5	293 (91)	120	
≥5.5	29 ( 9)	21	2.78 (1.74–4.42) <0.0001
<6.0	298 (93)	124	
≥6.0	24 ( 7)	17	2.54 (1.53–4.23) 0.0003
<6.5	302 (94)	128	
≥6.5	20 ( 6)	13	2.12 (1.20–3.76) 0.0100
<7.0	306 (95)	129	
≥7.0	16 ( 5)	12	2.84 (1.57–5.15) 0.0006

### Colorectal cancer-specific death

The cause of death could not be determined for 13 deceased patients, who were excluded, leaving 309 for analysis of colorectal cancer-specific death. Eighty-six patients died of colorectal cancer, 42 died of other causes and 181 remained alive at last contact. A competing risks regression model with NLR as the single, continuous covariate showed that the cumulative incidence of CRC-specific death increased as NLR increased (HR 1.07, CI 1.003-1.13, *p* = 0.038). However this association disappeared (HR 1.01, CI 0.92-1.12, *p* = 0.782) in a multivariable model into which NLR was forced (Table 5). The statistically significant covariates in this model were involvement of ≥ 40% of nodes, poor differentiation and infiltration of an adjacent structure or organ (Table 5). Because there was no significant independent association between NLR and cancer-specific survival, no attempt was made to find the optimal threshold for dichotomizing NLR in this context.

**Table 5:** Association between NLR, clinical and pathology features and death due to colorectal cancer with death due to other causes as a competing risk and association with death due to other causes with death due to colorectal cancer as a competing risk

	**Death due to CRC Bivariate association**	**Death due to CRC Multivariable association**	**Death due to other causes Bivariate association**	**Death due to other causes Multivariable association**
	**HR, (95% CI), Wald*p***	**HR, (95% CI), Wald*p***	**HR, (95% CI), Wald*p***	**HR, (95% CI), Wald*p***
Neutrophil/lymphocyte ratio	1.07 (1.003–1.13) 0.038	1.01 (0.92–1.12) 0.782	1.09 (1.02–1.17) 0.013	1.09 (1.03–1.15) 0.004
Male	0.74 (0.48–1.12) 0.154	--	1.91 (1.00–3.65) 0.051	2.32 (1.23–4.36) 0.009
Female				
Age ≥ 75 years	1.77 (1.15–2.72) 0.009	--	5.72 (2.98–10.95) <0.001	2.10 (1.03–4.30) 0.042
Age < 75 years				
Rectal tumor	0.89 (0.58–1.38) 0.602	--	1.05 (0.57–1.93) 0.870	--
Colonic tumor				
Tumor diameter ≥ 5cm	1.09 (0.71–1.67) 0.696	--	1.32 (0.72–2.42) 0.365	--
< 5cm				
Mucinous or signet ring	1.60 (0.90–2.84) 0.107	--	0.87 (0.30–2.49) 0.793	--
No				
Direct spread beyond muscularis propria	2.52 (1.19–5.33) 0.015	--	1.21 (0.52–2.78) 0.661	--
No				
Apical node involved	2.30 (1.23–4.27) 0.009	--	0.71 (0.22–2.30) 0.567	--
No				
≥ 4 nodes involved	2.27 (1.49–3.46) <0.001	--	1.14 (0.60–2.17) 0.691	--
No				
≥ 40% of nodes involved	3.18 (2.04–4.96) <0.001	2.91 (1.79–4.74) <0.001	0.93 (0.40–2.13) 0.863	
No				
Poorly differentiated	2.63 (1.68–4.11) <0.001	2.01 (1.24–3.26) 0.005	1.02 (0.48–2.15) 0.966	--
No				
Venous invasion	1.59 (0.97–2.62) 0.066	--	0.95 (0.44–2.05) 0.895	--
No				
Free serosal surface involved	2.76 (1.78–4.27) <0.001	--	1.07 (0.53–2.18) 0.846	--
No				
Adjacent structure infiltrated	4.56 (2.51–8.26) <0.001	5.06 (2.72–9.43) <0.001	0.31 (0.04–2.39) 0.262	--
No				
Postoperative chemotherapy	0.79 (0.51–1.21) 0.279	--	0.12 (0.05–0.26) <0.001	0.17 (0.07–0.39) <0.001
No				

The association between NLR and death was further examined in a regression model with non-CRC death classified as the event and death due to CRC as the competing risk (Table 5). Here, NLR was significantly and independently associated with non-CRC death (HR 1.09, CI 1.03-1.15, p = 0.004), along with male sex and age ≥ 75 years. Postoperative chemotherapy appeared to protect against death from other causes, presumably because such treatment was generally confined to “fit patients” who were not at great risk of non-cancer death.

The interpretation of these competing risk regression models is that NLR was not an independent prognostic factor for colorectal cancer death but was a prognostic factor for death due to other causes.

### Recurrence

Eleven deceased patients who had not had a recurrence and whose cause of death was unknown were excluded from analysis of recurrence because it was unknown whether their death was due to recurrent CRC. In the remaining 311 there were 108 recurrences: 16 local only, 84 systemic only and 8 with both local and systemic recurrence. Thirty-nine patients died without recurrence an 164 remained alive without known recurrence at last contact. The median time to recurrence was 15 months (range 1 – 60 months) and the follow-up time of censored patients who were not lost was 82 months (range 34 to 147 months). A regression model with non-CRC death as a competing risk showed no bivariate association between NLR and recurrence (HR 1.04, CI 0.97-1.11, p = 0.241, Table 6) and a multivariable model showed that ≥ 40% of nodes involved, poor differentiation, involvement of a free serosal surface and infiltration of an adjacent structure or organ were independently associated with recurrence but NLR, when forced into this model, was not (HR 0.99, CI 0.90-1.08, p = 0.772).

**Table 6:** Association between NLR, clinical and pathology features and any recurrence with death due to other causes as a competing risk

	**Number of patients**	**Number of recurrences**	**Bivariate association HR, (95% CI), Wald*p***	**Multivariable association HR, (95% CI), Wald*p***
Neutrophil/lymphocyte ratio	311	108	1.04 (0.97–1.11) 0.241	0.99 (0.90–1.08) 0.772
Male	171	56	0.81 (0.56–1.19) 0.291	--
Female	138	52		
Age ≥ 75 years	98	40	1.31 (0.88–1.94) 0.178	--
Age < 75 years	211	68		
Rectal tumor	124	46	1.09 (0.74–1.60) 0.676	--
Colonic tumor	185	62		
Tumor diameter ≥ 5cm	134	49	1.21 (0.82–1.77) 0.335	--
< 5cm	175	59		
Mucinous or signet ring	32	15	1.45 (0.87–2.42) 0.153	--
No	277	93		
Direct spread beyond muscularis propria	260	97	2.14 (1.15–4.00) 0.017	--
	49	11		
No				
Apical node involved	29	15	1.95 (1.10–3.45) 0.022	--
No	280	93		
≥ 4 nodes involved	98	47	1.89 (1.29–2.77) 0.001	--
No	211	61		
≥ 40% of nodes involved	56	32	2.36 (1.56–3.55) <0.001	1.87 (1.17–3.01) 0.009
No	255	76		
Poorly differentiated	64	34	2.09 (1.39–3.13) <0.001	1.66 (1.07–2.57) 0.024
No	247	74		
Venous invasion	61	26	1.50 (0.95–2.36) ).080	--
No	248	82		
Free serosal surface involved	73	41	2.66 (1.80–3.95) <0.001	1.83 (1.18–2.84) 0.007
No	236	67		
Adjacent structure infiltrated	22	16	3.86 (2.19–6.79) <0.001	2.93 (1.58–5.44) 0.011
No	287	92		
Postoperative chemotherapy	195	71	1.18 (0.79–1.77) 0.407	--
No	114	37		

## Discussion

In this study of patients with stage C CRC, increasing preoperative NLR was independently associated with diminished overall survival after adjustment for other independent prognostic variables. Findings on overall survival are inconsistent in other reports. Only Hung et al. have shown results equivalent to ours, albeit in patients with stage II colon cancer [9]. Walsh et al. [7] and Kwon et al. [4] found a bivariate but no multivariable association between NLR and overall survival in patients with stages I to IV tumor whereas Leitch et al. found neither in a similar patient pool [10]. Clearly overall survival is an important outcome for patients as it encompasses potential mortality arising both from the CRC itself and from treatment as well as from other causes which may be tangentially associated (e.g. hospital-acquired infection or other iatrogenic illness). Furthermore, the prime concern for patients is how long they survive, not what causes their death. It is striking that these different studies have yielded such inconsistent results on the association between NLR and overall survival; however this may be arise from the mixed nature of these other populations, particularly in terms of tumor stage. There are recent data that have linked NLR levels with outcomes following coronary events and procedures and these data might be indicative of the types of non-cancer related problems that could be influencing non-cancer induced mortality in this and other cohorts [27]. Traditionally, assessments of performance status have been linked to survival outcomes in cancer and it is possible that inflammatory markers might provide a less subjective equivalent of performance status. In previous studies from our group, inflammatory markers have correlated well with performance status scores [28]. The relatively poor (though statistically significant) correlation between NLR and OS could have been influenced by the fact patients coming to surgery tend to be relatively fit, therefore reducing number of unwell patients who would be more likely to have high NLR levels. Furthermore, in our practice, the proportion of patients having emergency surgery is very low.

Some authors have reported on the outcomes of CRC-specific survival [5,7,10] or disease -free survival [6,9,11] or recurrence free survival [12], presumably because it is believed that these outcomes will enable a more specific assessment of the link between NLR and response to CRC. Results for CRC-specific survival are no more consistent than for overall survival; Leitch et al. found no association with NLR, [10] Walsh et al. found a bivariate but no multivariable association [7], and Liu et al. found a multivariable but no bivariate association [5]. All three of these studies have the technical problem that the survival times of patients who died of causes other than CRC were censored at the date of death, which would lead to an inflated estimate of the incidence of death due to CRC [13,14]. This arises because, in standard time-to-event analysis, it is assumed that all censored patients remain equally at risk of death due to CRC after the time of last contact but this of course is incorrect for patients who die of another cause. The solution to this problem is to use competing risks Cox regression in which patients who die of causes other than the cause of interest are not censored but instead removed from the calculations at the time of death. If the proportion of all deaths due to other causes is either very large or very small, use of the competing risks method may be irrelevant; in other circumstances competing risks regression is both appropriate and necessary. In our study 33% of all deaths were due to causes other than CRC and competing risks regression showed no statistically significant association between NLR and CRC-specific mortality after adjustment for other prognostic factors. On the other hand, NLR was shown to be an independent prognostic factor for deaths due to other causes.

The outcome of recurrence-free survival was examined in one study [12] and disease-free survival in three [6,9,11], though in no case were these defined explicitly in terms of which patients had censored survival times. This is important because various and sometimes contradictory definitions of these concepts and of time to recurrence exist [29] and the results of Kaplan-Meier and Cox regression analyses of recurrence will vary, depending on the definition used. These definitional complexities are avoided by using competing risk methods in which patients who have a recurrence are coded as failures, those who are lost or remain alive without recurrence are censored and those who die of other causes are deemed to experience a competing risk.

Ours is the first report to use the contemporary statistical method of competing risks Cox regression to analyse the association between NLR and CRC-specific survival and recurrence. With this method we found no association, either bivariate or multivariable, between NLR and either death due to CRC or recurrence.

Most other studies of NLR in primary CRC have dichotomized NLR at < 5 versus ≥ 5 [4,6,7,9,10] as proposed by Zahorec [8], although a cutting point at < 2 versus > 2 (*sic*) was used by Liu et al. because this was approximately the upper limit in their normal control patients [5]. ROC analysis was used to set the threshold in two studies, producing NLR cutting points of ≤ 4 versus > 4 [12] and ≤ 3 versus > 3, [11] in both cases resulting in the conclusion that NLR was an independent prognostic factor for poor outcome. Our own attempt at using ROC analysis to determine an optimal for NLR prediction of overall survival was equivocal; no single, clear-cut solution could be found. This was because the association between NLR and survival, albeit statistically significant, was weak. We concluded that, while NLR is a weak independent predictor of overall mortality after resection of stage C CRC, this is because it predicts death from other causes, not because it predicts recurrence or cancer-related death. As the weak association between NLR and overall mortality prohibits a clear-cut differentiation of NLR into “high” and “low” ranges of values, the sensitivity and specificity of any such split will be poor and the positive and negative predictive values will be close to 50%, meaning that such a test would have no clinical value. Despite the optimistic conclusions of Chiang et al. [11] and Ding et al. [12] regarding their analyses, in both cases their ROC curves depart only slightly from the null diagonal over a wide range of sensitivities, clearly showing that the chosen optimum cutting points give poor differentiation between “high” and “low” values of NLR and hence would yield very poor sensitivity, specificity and predictive values if the authors had given all of these measures. A further reservation about the use of ROC curves in this context is that the outcome variable conventionally used is simply the crude death rate (or crude recurrence) which ignores both the censoring of survival times and the problem of competing risks regarding CRC-specific survival or recurrence.

An important issue is the particular pool of patients in which the NLR has been investigated.

Some studies include stage I patients [4-7,10] which is unlikely to be productive because of the almost universally good prognosis in such patients. The same studies also include stage IV patients despite their already known very poor prognosis. Although the NLR is unlikely to have any prognostic importance in stage IV it is possible that it may have predictive significance for outcomes from chemotherapy in such patients [17] and further investigations appear justifiable. It is likely that in stage IV patients there is a greater prevalence of cancer-associated inflammation than in stage III patients simply because of the widespread nature of their disease. Hence, it is possible that high levels of NLR are more likely to predict overall poor outcome in advanced cancer patient populations and therefore assist in the more objective selection of patients to receive or not to receive palliative chemotherapy. We had hoped that NLR levels might assist in defining groups more likely to benefit from adjuvant chemotherapy in patients with stage III cancers; however this has not been demonstrated in the current cohort.

## Conclusion

In a large pool of well-documented patients who had a resection for stage C CRC and using contemporary competing risks survival methods we found that an elevated NLR was independently associated with diminished overall survival. However there was no association between NLR and either tumor recurrence or CRC-specific death but elevated NLR was independently associated with non-cancer death. Thus the link between NLR and overall mortality was not specific to colorectal cancer but apparently arose because patients with an elevated inflammatory status preoperatively were likely to progress to earlier death, but not necessarily because of their cancer.

## Abbreviations

NLR: Neutrophil/lymphocyte ratio; CRC: Colorectal cancer; ROC: Receiver operating characteristic; mGPS: Modified Glasgow Prognostic Score; CRP C: Reactive protein.

## Competing interests

None of the authors have any financial or non-financial competing interests in relation to this paper.

## Authors’ contributions

LJ carried out acquisition and interpretation of data, analysis and made contribution to study design. OD developed methodology and analysed the data. LJ and OD drafted the paper. CC and PC contributed to the critical review of the paper. SC contributed to the conception and design of the study and was involved in general critical review of the work. All authors read and approved the final manuscript.

## Pre-publication history

The pre-publication history for this paper can be accessed here:

http://www.biomedcentral.com/1471-2407/13/442/prepub
